# The Function of Autophagy in Lace Plant Programmed Cell Death

**DOI:** 10.3389/fpls.2019.01198

**Published:** 2019-10-22

**Authors:** Adrian N. Dauphinee, Georgia L. Denbigh, Alice Rollini, Meredith Fraser, Christian R. Lacroix, Arunika H. L. A. N. Gunawardena

**Affiliations:** ^1^Department of Biology, Dalhousie University, Halifax, NS, Canada; ^2^Department of Molecular Sciences, Swedish University of Agricultural Sciences, Uppsala, Sweden; ^3^Department of Biology, University of Prince Edward Island, Charlottetown, PE, Canada

**Keywords:** programmed cell death (PCD), autophagy, TEM, confocal microscopy, immunolocalization, ATG8, leaf development, perforation formation

## Abstract

The lace plant (*Aponogeton madagascariensis*) is an aquatic monocot that utilizes programmed cell death (PCD) to form perforations throughout its mature leaves as part of normal development. The lace plant is an emerging model system representing a unique form of developmental PCD. The role of autophagy in lace plant PCD was investigated using live cell imaging, transmission electron microscopy (TEM), immunolocalization, and *in vivo* pharmacological experimentation. ATG8 immunostaining and acridine orange staining revealed that autophagy occurs in both healthy and dying cells. Autophagosome-like vesicles were also found in healthy and dying cells through ultrastructural analysis with TEM. Following autophagy modulation, there was a noticeable increase in vesicles and vacuolar aggregates. A novel cell death assay utilizing lace plant leaves revealed that autophagy enhancement with rapamycin significantly decreased cell death rates compared to the control, whereas inhibition of autophagosome formation with wortmannin or blocking the degradation of cargoes with concanamycin A had an opposite effect. Although autophagy modulation significantly affected cell death rates in cells that are destined to die, neither the promotion nor inhibition of autophagy in whole plants had a significant effect on the number of perforations formed in lace plant leaves. Our data indicate that autophagy predominantly contributes to cell survival, and we found no clear evidence for its direct involvement in the induction of developmental PCD during perforation formation in lace plant leaves.

## Introduction

Autophagy is a major catabolic pathway critical for the survival of eukaryotes as it enables cells to maintain homeostasis under stressful conditions such as nutrient deprivation or starvation (Klionsky et al., 2016). Autophagy plays a central role in many processes including programmed cell death (PCD), stress responses, and longevity (Floyd et al., 2015). It has been proposed that there are three classes of autophagy in plants: i) microautophagy which involves the direct passing of contents into a lytic vacuole; ii) macroautophagy is coordinated by evolutionarily conserved AuTophaGy-related (ATG) proteins and involves either the bulk or selective sequestration of cytoplasmic cargoes into double-membrane vesicles known as autophagosomes, which are then delivered to a lytic compartment for degradation; and iii) mega-autophagy, defined by cellular degradation following the release of hydrolases from the vacuole after tonoplast rupture (Van Doorn and Papini, 2013; Marshall and Vierstra, 2018). Of these classes, macroautophagy, hereafter autophagy, is the only well-characterized form of autophagy in plants (Batoko et al., 2017) and is therefore the focus of this study.

**Figure 1:**
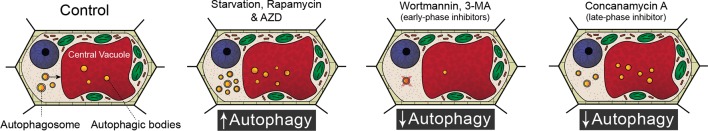
Modulating autophagic flux. Compared to standard control conditions, starvation, rapamycin, and AZD 8055 increase the number of autophagosomes within a cell. Wortmannin and 3-methyladenine (3-MA) disrupt membrane formation and are therefore early phase inhibitors of autophagy. Concanamycin A inhibits the breakdown of autophagic bodies and cargoes in the vacuole.

Because of the significant involvement of autophagy in a wide range of developmental processes and stress responses, there has been a substantial effort to identify chemicals that modulate autophagic flux (**Figure 1**). Autophagy is an evolutionary conserved process in fungi, plants, and animals. In fact, a great deal of our understanding of the regulatory genes involved in autophagy originated from yeast (*Saccharomyces cerevisiae*) mutagenic screens (Tsukada and Ohsumi, 1993). There have been 40 ATG proteins identified in yeast to date, and among them is ATG8, which is a ubiquitin-like protein integral for autophagosome membrane formation (Shpilka et al., 2011; Avin-Wittenberg et al., 2018; Marshall and Vierstra, 2018). The central regulator of autophagy is the target of rapamycin (TOR) kinase, which comprised two complexes: TORC1 and TORC2 (Liu and Bassham, 2012). Autophagy is inhibited by TOR, and therefore compounds such as rapamycin (Ballou and Lin, 2008) and AZD 8055 (Din et al., 2012), which block TOR, lead to an increase in autophagic activity. A similar effect can be achieved through starvation, which also inhibits TOR (Kwak et al., 2012; Liu and Bassham, 2012; Heras-Sandoval et al., 2014). Autophagy can also be inhibited with compounds such as wortmannin or 3-methyladenine that interfere with vesicle nucleation by inhibiting phosphoinositide 3-kinase (Klionsky et al., 2016; Marshall and Vierstra, 2018). Additionally, autophagy can be inhibited indirectly toward the end of autophagic flux by halting the breakdown of autophagic bodies *via* raising the vacuolar pH through the specific inhibition of vacuolar ATPases with concanamycin A (Huss et al., 2002).

**Figure 2:**
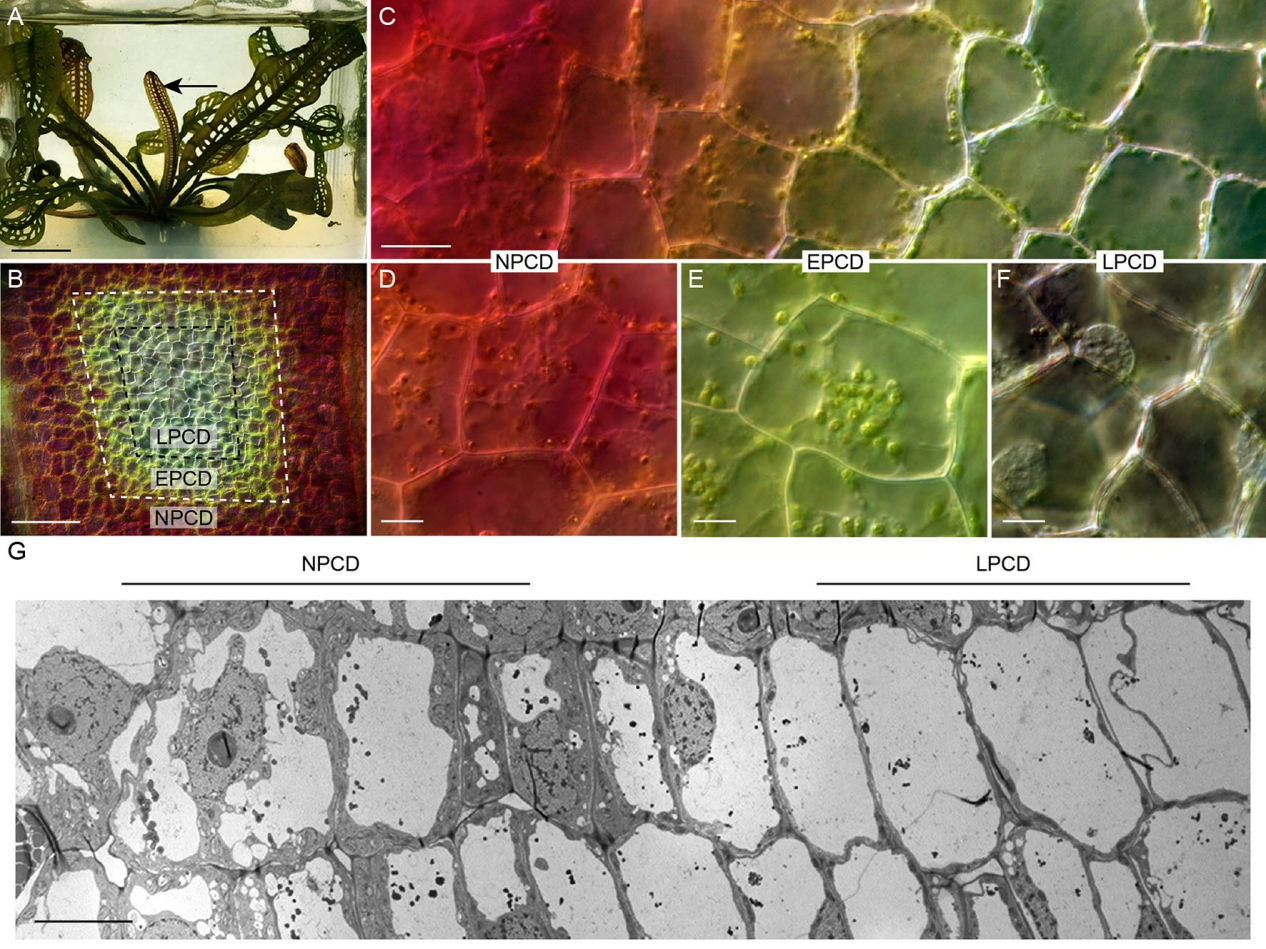
The lace plant programmed cell death (PCD) model system. **(A)** Lace plant grown in axenic magenta box culture producing a window stage leaf (arrow) where PCD is actively occurring. Between the longitudinal and transverse veins is the areole **(B)**, and in the window stage of leaf development, there is a gradient of cell death **(C)**. **(D**–**F)** Higher magnification of representative cells along the gradient of cell death. **(D)** Non-PCD (NPCD) cells do not die during perforation formation. **(E)** Early-PCD (EPCD) cells have lost anthocyanin pigmentation and are undergoing PCD. **(F)** Late-PCD (LPCD) cells are nearly devoid of pigmentation and are near death. **(G)** Merged transmission electron microscopy (TEM) micrographs of the lace plant gradient of PCD. Scale bars: A = 1 cm; B = 80 µm; C = 20 µm; D–F = 10 µm; G = 20 µm.

The lace plant (*Aponogeton madagascariensis*) is an aquatic monocot with a unique perforated morphology created by developmentally regulated PCD (**Figure 2A**; Gunawardena et al., 2004). The lace plant is an emerging model for studying PCD due to the predictability of perforation formation, its nearly transparent leaves that facilitate live cell imaging, and established sterile cultures for *in vivo* pharmacological experimentation (Gunawardena et al., 2006). The first visible sign that PCD is underway is the disappearance of anthocyanins (which are potent antioxidants) between longitudinal and transverse veins in spaces known as areoles (Gunawardena et al., 2004). The disappearance of anthocyanins provides a visual gradient of PCD within each areole (**Figures 2B, C**): non-PCD (NPCD; **Figure 2D**) cells retain anthocyanins throughout perforation formation; early-PCD (EPCD; **Figure 2E**) cells have lost anthocyanin and are fated to die but still have an abundance of chlorophyll pigmentation; and cells that are in the late-PCD (LPCD; **Figure 2F**) are mostly devoid of pigmentation and near death (Lord et al., 2011; Dauphinee et al., 2017). When observed with transmission electron microscopy (TEM), the PCD gradient highlights the degradation of LPCD compared to NPCD cells (**Figure 2G**). The dynamics and time-course analysis of lace plant PCD has been described in detail (Wertman et al., 2012), and preliminary evidence suggested that autophagy may be involved; however, its function in lace plant PCD remains unknown. Autophagy has been implicated in the regulation of various plant PCD systems and therefore warrants further investigation in lace plants. The purpose of this study is to elucidate the function of autophagy in developmental PCD during lace plant leaf development.

## Materials and Methods

### Plant Material and *in Vivo* Experiments

Lace plant (*A. madagascariensis*) cultures were propagated according to Gunawardena et al. (2006). To test the effects of autophagy modulators on the formation of perforations, 40 ml septum-lidded vials were used (Sigma-Aldrich) according to Dauphinee et al. (2012). Plants were grown in magenta boxes under daylight deluxe fluorescent lighting (Phillips) on 12-h dark-light cycles at an intensity of 125 μmol m^−2^ s^−1^ for approximately 4 weeks. They were then transferred to the vials and allowed to acclimate for 1 to 2 weeks. Once plants produced two to three perforated leaves, they were assigned randomly to a treatment group. Treatments were applied once to the liquid media and were dissolved in dimethyl sulfoxide (DMSO). The autophagy modulator treatments were optimized using a gradient of concentrations. The mock control treatment group received an equal volume of DMSO used for the autophagy modulator treatments. Optimal concentrations had no severe effects on leaf growth or showed signs of stress, which was observed at higher concentrations with the autophagy modulators. The optimized concentrations included 5 µM rapamycin (Enzo Scientific, BML-275), 1 µM AZD 8055 (AZD; ApexBio Technology, A8214), and 5 µM wortmannin (Cayman Chemical, 10010591).

### Autophagy Modulation

Autophagy modulating compounds were also used for live cell imaging (described below) of detached window stage leaves. Autophagy modulation was achieved using the following treatments: distilled water (16 h starvation), 5 µM rapamycin, 1 µM AZD, 5 µM wortmannin, and 1 µM concanamycin A (Santa Cruz Biotechnology, sc-202111). For the cell death assay, leaves were mounted in treatment solution and observed continuously (video capture) for 6 h unless stated otherwise. Treatment times for the autophagy modulators were 3 h for TEM and live cell imaging experiments. The mock control treatment group received an equal volume of DMSO (BioShop Canada, DMS666).

### Cell Death Assay and Live Cell Imaging

Window stage leaves were detached under sterile conditions and kept in distilled water for 16 h. Leaves were mounted in the designated treatment solution and placed on a custom grooved slide as per Wertman et al. (2012). The slide was then sealed with VALAP (a mixture of VAseline, LAnolin, and Paraffin wax) according to Kacprzyk et al. (2015). Videos were then captured on a Nikon Eclipse 90i microscope fitted with a DXM1200C digital camera using the audio video interleave recording function of NIS Elements AR 3.1 software (Nikon Instruments). Experiments ran for a maximum of 6 h or until all LPCD stage cells collapsed. A minimum of six independent replicates were carried out for each treatment. The number of dead cells (collapsed PMs) were counted prior to the beginning of the experiment and at the end of the observation period to determine the death rate per hour. Evans blue (Sigma-Aldrich, 46160) staining was used to facilitate the counting of dead cells at the end of the experiments and carried out according to Wertman et al. (2012).

### ATG8 Immunolocalization

Intracellular detection of ATG8 was achieved in lace plant leaves using an adapted immunolocalization protocol (Pasternak et al., 2015; Mishra et al., 2017). Four independent replicates were carried out using window stage leaves taken from axenic cultures that were then rinsed gently with distilled water and fixed in 100% methanol at 37°C for 30 min. The tissues were then transferred to 800 µl of fresh 100% methanol and hydrophilized to a concentration of 20% methanol at 60°C through the addition of 200 µl of distilled water every 2 min for 32 min. Leaves were then cut into 2-mm^2^ pieces and rinsed in distilled water before being placed onto a multiwell slide. The leaf pieces were then allowed to dry on the slide for approximately 5 min until all excess liquid evaporated. Blocking was performed for 30 min at 37°C with 4% (w/v) low-fat milk in 1X MTSB (microtubule stabilization buffer: 7.5 g Pipes, 0.85 g EDTA, 0.61 g MgSO_4_*7H_2_O, and 1.25 g KOH, pH 7). Incubation with the ATG8 rabbit polyclonal primary antibody (Agrisera, AS14 2769) was done at 37°C for 30 min at a 1:1,000 dilution in MTSB. Negative controls were incubated with ATG8 preimmune serum (Agrisera, AS14 2769PRE) under the same conditions. Samples were then rinsed three times for 5 min each with MTSB. Secondary incubation was done with a 1:2,000 dilution of goat anti–rabbit Dylight® 488 polyclonal antibody (Agrisera, AS09 633) in MTSB. The samples were then rinsed as above and mounted in Mowiol (Sigma, 9000-89-5) prior to scanning with a Nikon Eclipse *Ti* C1 confocal system (Nikon). Z-stack images were analyzed and converted to maximum intensity projections using NIS Elements AR 3.1 software; fluorescent punctate structures (puncta) were counted automatically using ImageJ (particle analysis set to a lower brightness threshold of 75), and a central focal plane of the transmitted light channel was used to approximate the number of cells in the field of view. The data were normalized to the mean number of puncta in NPCD cells and expressed as the relative number of ATG8-positive puncta per cell.

### ATG8 Immunoblotting

Three window stage leaves from sterile cultures were blot dried and had their midribs removed prior to freezing in liquid nitrogen. Tissues were macerated on ice in equal volumes of Pipes buffer (pH 6.8) and a protease inhibitor solution. The protease inhibitor solution consisted of a 1:2 ratio of two components (Component A: 10 mg/ml leupeptin and 10 mg/ml soybean trypsin inhibitor dissolved in Pipes buffer; Component B: 10 mg/ml pepstatin and 20 mg/ml phenylmethylsulfonyl fluoride dissolved in 95% ethanol). Following maceration, the samples were centrifuged for 15 min at 16,000 *g*. Total protein concentration of the supernatant was determined using the Bradford assay. A 1:1 mixture of sample to 2X Laemmli Buffer (Bio-Rad, 1610737) with 5% β-mercaptoethanol (v/v) was prepared prior to gel electrophoresis. A total of 10 µg of protein was loaded for each sample lane, along with 5 µl of the Precision Plus Protein Standards solution (Bio- Rad, 1610374) in a 8% to 16% sodium dodecyl sulfate (SDS) polyacrylamide Mini-PROTEAN TGX precast gel (Bio- Rad, 456- 1103). The gel was resolved at 100 V for 2 h in ice-cold running buffer (0.1% SDS [v/v], 25 mM Tris, and 192 mM glycine, 8.3 pH). Protein transfer to a 0.2 µm nitrocellulose membrane (Bio-Rad, 1610112) occurred overnight at 120 mA in a transfer buffer (20% methanol [v/v], 25 mM Tris, and 192 mM glycine, 8.3 pH) at room temperature.

Ponceau staining on the membrane was done for 5 min to confirm successful protein transfer. The membrane was then rinsed for 2 min in TBS-T (10 mM Tris, 140 mM NaCl, Tween-20, pH 7.4) prior to being scanned. The membrane was blocked in a 5% (w/v) low-fat milk powder TBS-T solution with mild shaking and then incubated overnight with the ATG8 primary antibody (detailed above) at a 1:10,000 dilution in TBS-T with 3% low-fat milk powder (w/v) at 2°C. The following day, the membrane was rinsed with mild shaking at 1-, 2-, and 3-min intervals in TBS-T. The secondary antibody (horseradish peroxidase–conjugated goat–anti-rabbit polyclonal; AS609 602, Agrisera) was applied at a 1:20,000 dilution in TBS-T for 30 min, and then the membrane was rinsed as described above with an additional 2-min rinse in Tris-buffered saline (10 mM Tris, 140 mM NaCl, 0.1%, pH 7.4). Clarity Western ECL substrate (Bio-Rad, 1705061) was used according to the manufacturer’s instructions. Protein bands were resolved using an MF-ChemiBIS 3.2 gel documentation system (DNR Bio-Imaging).

### Acridine Orange and Monodansylcadaverine Staining

Window stage leaves were taken from cultures and had their midrib removed prior to staining. The leaves were then cut into 2-mm^2^ pieces prior to being placed in 30 µM acridine orange or a combination of acridine orange and 300 μM monodansylcadaverine (MDC) dissolved in phosphate-buffered saline. The leaf pieces were incubated at room temperature on a rotary shaker for 2 h at room temperature prior to being rinsed three times for 5 min with distilled water. Samples were mounted in distilled water and then imaged with confocal microscopy. Acridine orange was excited at 488 nm and detected at 525/25 nm (green) and 595/50 nm (red). Excitation of MDC was achieved with 405-nm light, and the emission filters for the dual stain experiments included 450/35 nm (blue), 525/25 nm (green), and 595/50 nm (red). Cyan, green, and magenta pseudocolors were applied for detecting blue, green, and red fluorescence, respectively, using ImageJ.

### Transmission Electron Microscopy

Window stage lace plant leaves were taken from sterile cultures and treated (four independent replicates) for 3 h prior to having their midribs removed and sectioned into 2-mm^2^ pieces. The leaf pieces were then fixed for a minimum of 2 h with 2.5% solution of glutaraldehyde in a 0.1 M sodium cacodylate buffer. The samples were then rinsed three times for 10 min each time, with the 0.1 M sodium cacodylate buffer. Secondary fixation with 1% osmium tetroxide was done for 48 h under vacuum (20 psi). The samples were then rinsed with distilled water briefly before being placed in 0.25% uranyl acetate at 4°C overnight. The samples were then dehydrated through a graduated series of acetone at 50%, 70%, 70%, 95%, 95%, 100%, and 100% for 10 min at each step of the process. Epon-Araldite resin was used to infiltrate the samples, initially in a 3:1 ratio of 100% acetone to resin for 3 h. This step was followed by transferring the samples to a 1:3 ratio of 100% acetone and resin overnight. The samples were then placed in 100% Epon-Araldite resin for 6 h, with the solution being refreshed once during that time. The embedded samples were then cured for 48 h at 60°C. Thin sections were cut using an Ultracut E Ultramicrotome (Reicher-Jung) with a diamond knife (100-nm thickness) and placed on formvar/carbon support film copper grids (Cedarlane, FCF205-CU-25). Staining was done using 2% aqueous uranyl acetate for 10 min, followed by two 5-min rinses with distilled water, 4 min in lead citrate counterstain, and then a final quick rinse with distilled water. The samples were viewed with a JEM 1230 Transmission Electron Microscope (JEOL) at 80 kV, and images were captured using a Hamamatsu ORCA-HR digital camera.

### Statistical Analysis and Data Representation

Data analysis and graphical representations used GraphPad Prism 5 software (GraphPad Software Inc.). Data are represented as mean ± standard error. Maximum intensity projections of confocal z-stacks were made using NIS Elements AR 3.1 software. Figures were prepared using Adobe Illustrator and Adobe Photoshop, and videos were assembled with Premiere Pro (CC; Adobe Systems Inc.). When necessary to improve clarity, adjustments to brightness, contrast, and exposure were made consistently with all replicates.

## Results

### The Involvement of Autophagy in Lace Plant Developmental PCD

**Figure 3:**
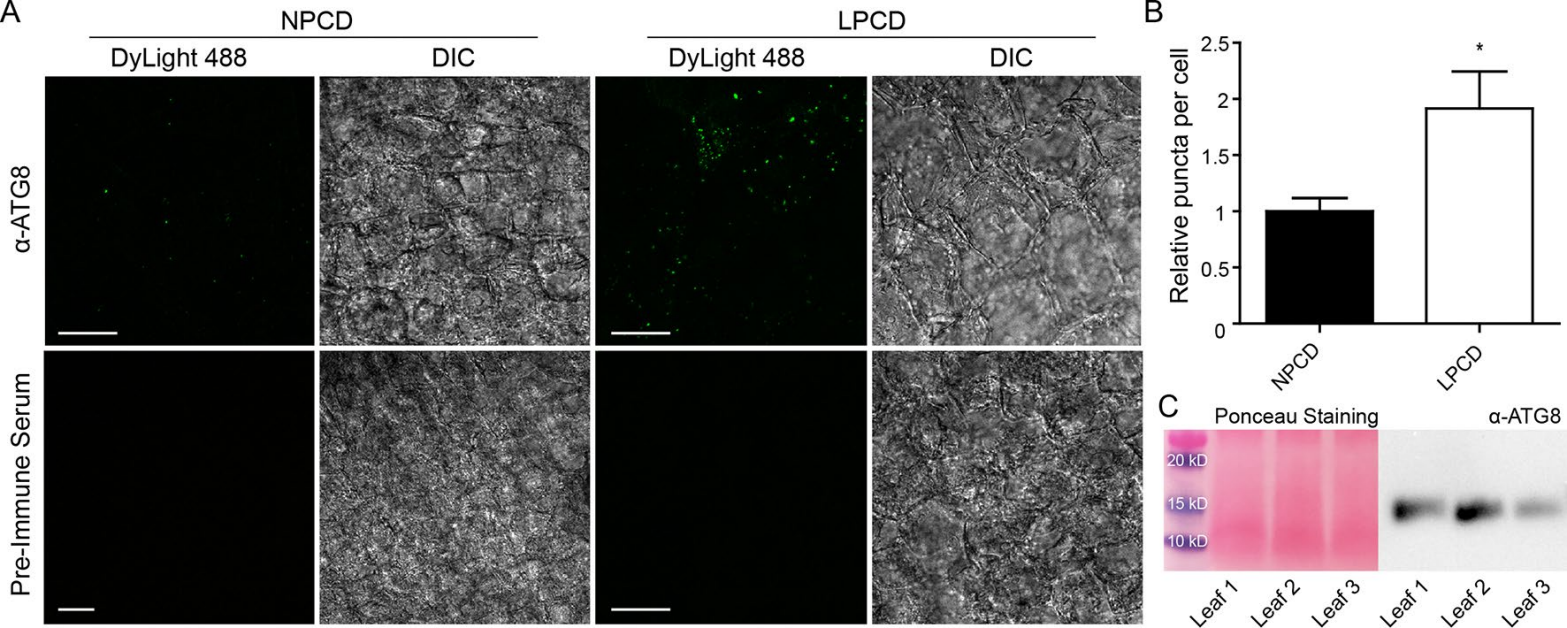
**(A)** Nonprogrammed cell death (NPCD) and late-PCD (PCD) cells following immunostaining with α-ATG8 and DyLight 488. Fluorescent images represent maximum intensity projections and corresponding differential interference contrast (DIC) images are from a representative optical section. **(B)** Relative number of ATG8-positive puncta per cell for NPCD and LPCD cells (normalized to NPCD). n = 4 leaves from individual plants, Student *t* test, *P < 0.05, error bar represents standard error. **(C)** Ponceau-stained membrane and corresponding immunoprobing for ATG8 in window stage leaves (n = 3) from individual plants. Scale bars: 10 µm.

**Figure 4:**
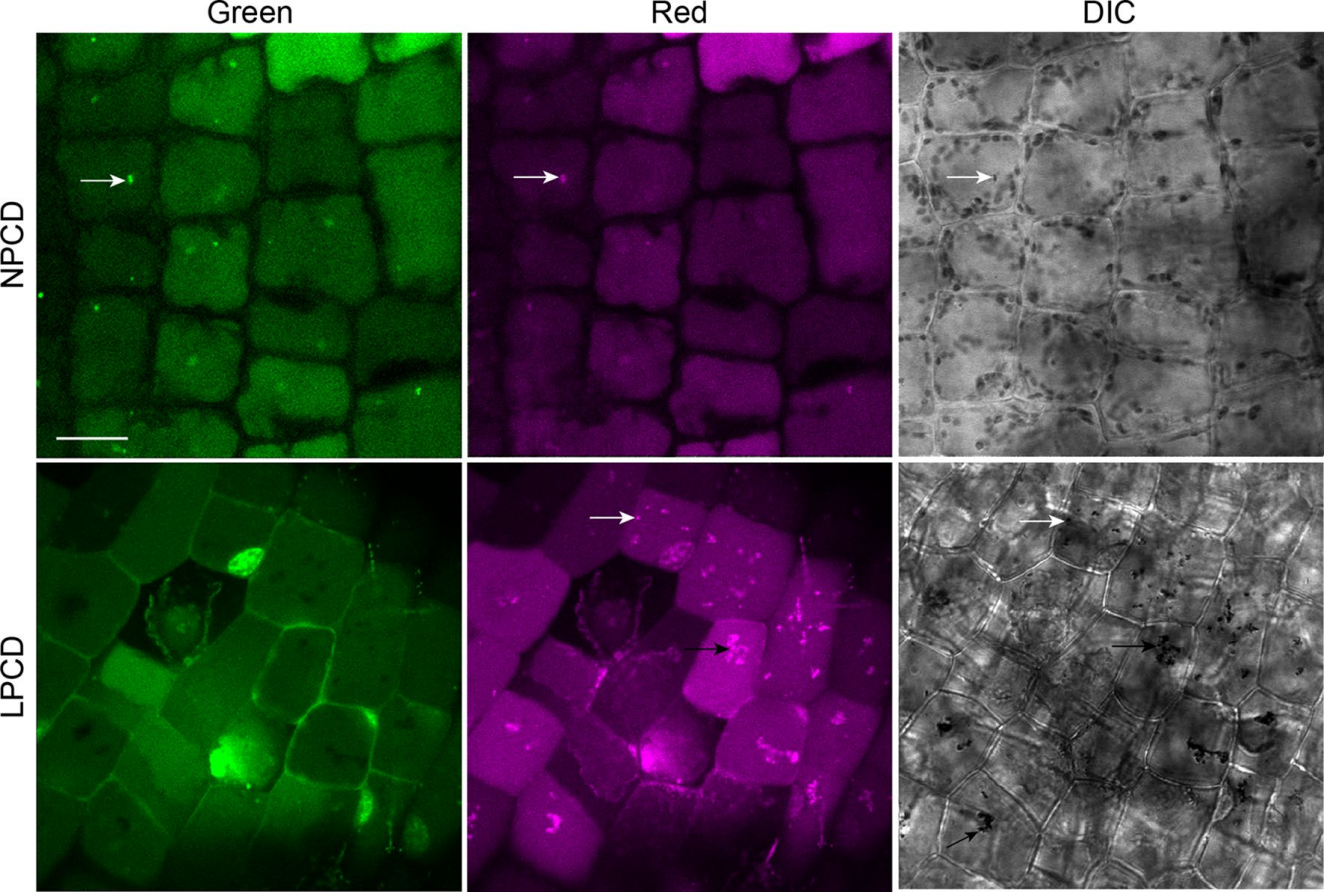
Acridine orange staining in window stage leaves. Nonprogrammed cell death (NPCD) and late-programmed cell death (LPCD) stage cells were stained with 30 µM acridine orange. Fluorescent puncta were found in both cell types (white arrows), and large vacuolar aggregates were seen in LPCD cells (black arrow). Scale bar: 20 µm.

To assess whether autophagy occurs during lace plant PCD, immunostaining of NPCD and LPCD cells in fixed window stage leaves was carried out with ATG8 and DyLight 488 antibodies and a negative control with the α-ATG8 preimmune serum (**Figure 3A**). Both healthy (NPCD) and dying (LPCD) lace plant cells contained ATG8-positive puncta; however, there was a significant increase in puncta in LPCD cells (**Figure 3B**). Immunoblotting for ATG8 was also carried out using protein extracts of window stage leaves to verify ATG8 antibody binding in lace plant samples (**Figure 3C**). The lysotropic dye acridine orange was also used to compare autophagy in living NPCD and LPCD cells of window stage leaves (**Figure 4**; **Supplementary File 1**). There were fluorescent puncta in both NPCD and LPCD cells (white arrows, **Figure 4**). However, there were considerably more puncta in LPCD cells that had more red than green fluorescence. Vacuolar aggregates in LPCD cells were also positive for acridine orange staining (black arrow, **Figure 4**). Monodansylcadaverine and acridine orange dual staining was also performed in NPCD cells and revealed a similar staining pattern (**Supplementary File 2**).

### Autophagy Modulation and Live Cell Imaging

**Figure 5:**
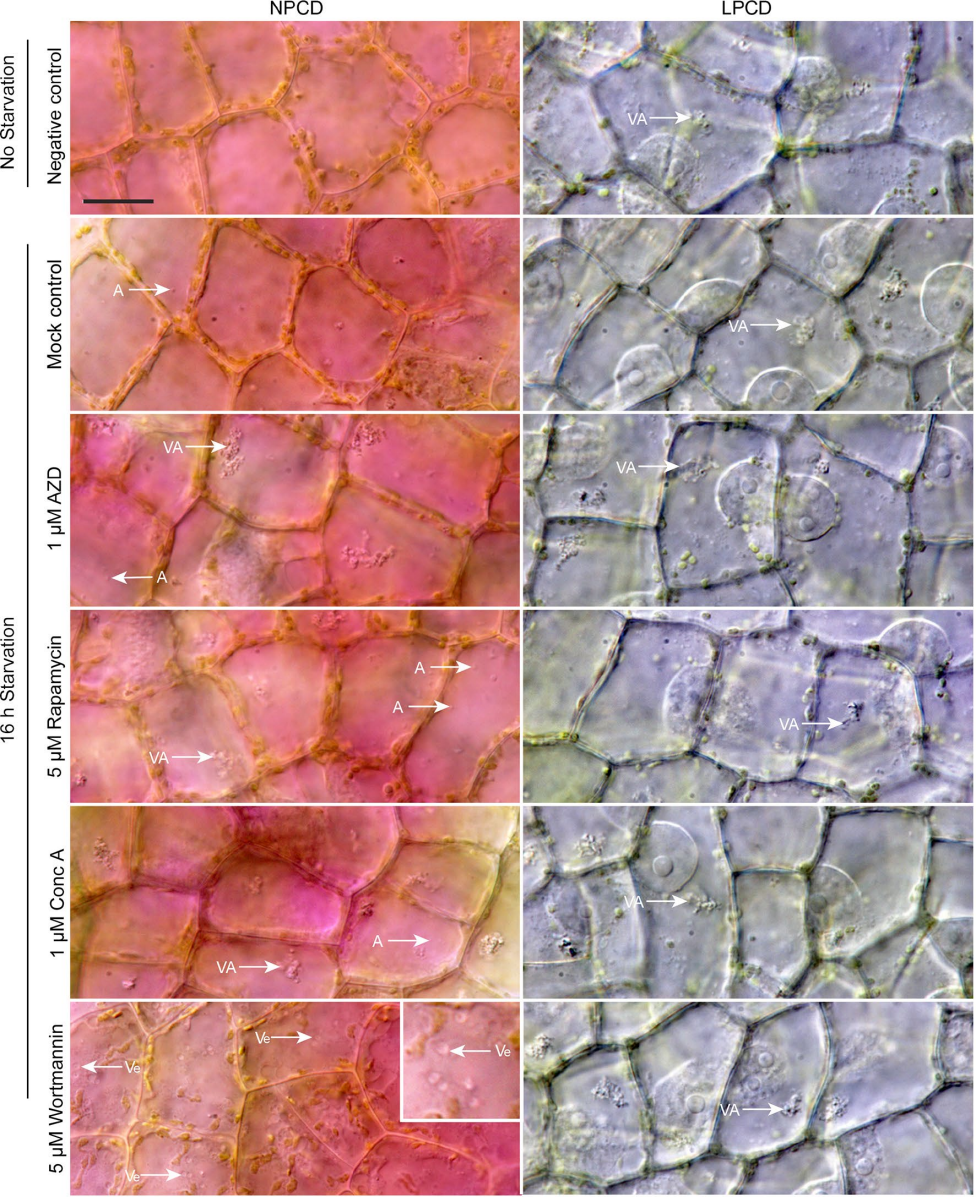
Live cell imaging of non-programmed cell death (NPCD) and late-PCD (LPCD) cells in window stage leaves exposed to various conditions. The negative control was a sample taken directly from plants growing in the culture media. All other treatment groups were subjected to a 16-h starvation period in distilled water prior to 3-h exposure to one of the following: 0.05% DMSO (mock control), 1 µM AZD 8055 (AZD), 5 µM rapamycin, 1 µM concanamycin A (Conc A), or 5 µM wortmannin. VA = vacuolar aggregates, A = autophagosome-like vesicle, inset displays large, organelle-containing vesicles (Ve). n = 3 leaves from individual plants. Scale bar: 20 µm. For additional information see **Supplementary Files 3** and **4**.

Live cell imaging was used to determine the effects of autophagy modulation on NPCD and LPCD cells of lace plant window stage leaves (**Figure 5**; see also **Supplementary Files 3** and **4**). The negative control group represents leaves taken directly from culture, whereas all other treatment groups had a 16-h starvation period prior to a 3-h exposure to an autophagy modulator. Qualitative assessment of the micrographs and the corresponding **Supplementary Videos 3** and **4** suggest that autophagy modulation with 1 µM AZD, 5 µM rapamycin, and 1 µM concanamycin A led to the formation of large vacuolar aggregates (VA, **Figure 5**) and numerous small, spherical autophagosome-like vesicles (A, **Figure 5**) that were most distinguishable with time-lapse imaging (**Supplementary Files 3** and **4**). Wortmannin treatment also resulted in the formation of large vesicles in NPCD cells that appeared to contain organelles (Ve, NPCD, **Figure 5**; **Supplementary File 3**).

### Ultrastructural Analysis

**Figure 6:**
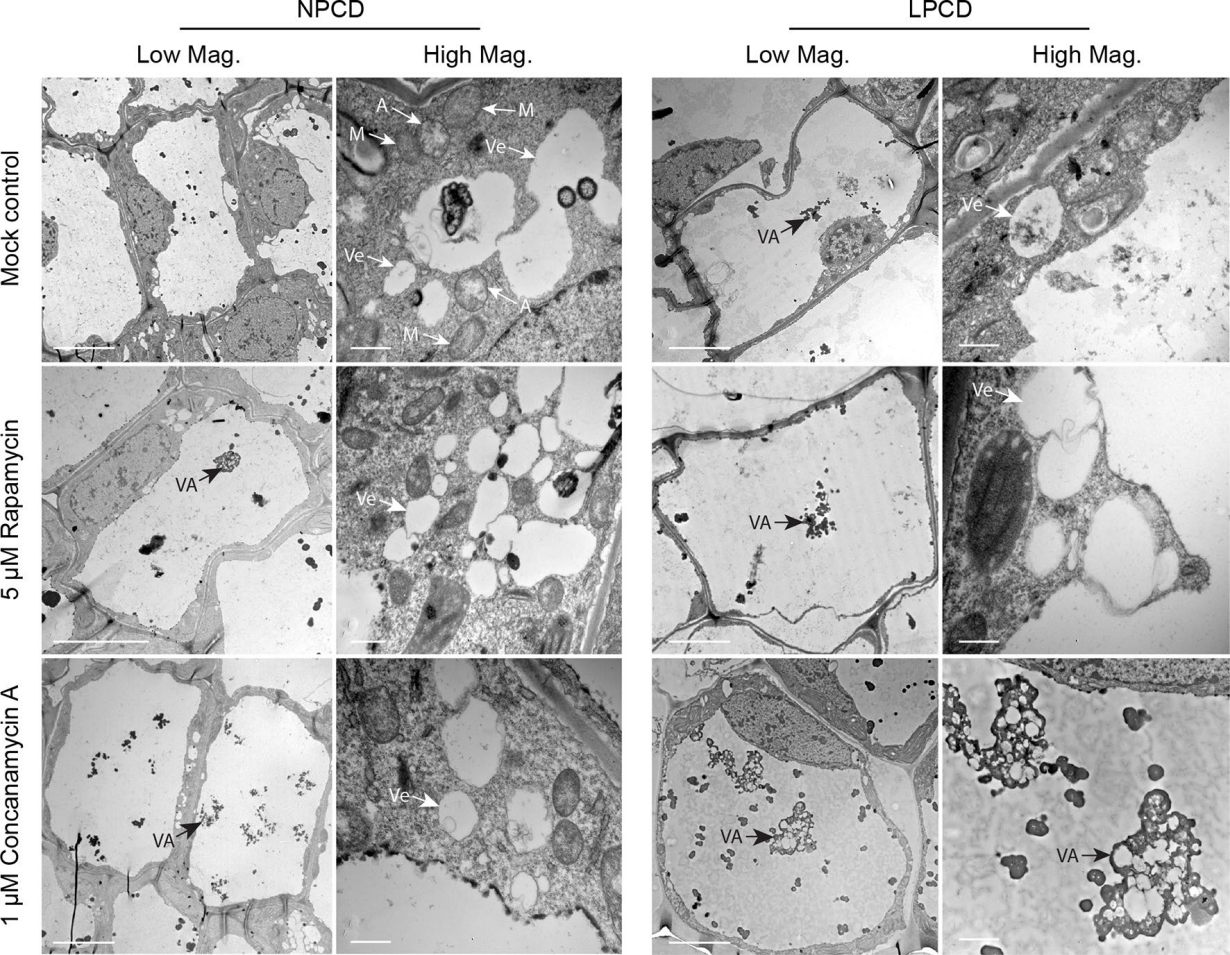
Non-programmed cell death (NPCD) and late-PCD (PCD) cells treated with 0.05% DMSO (mock control), 5 µM rapamycin, or 1 µM concanamycin A. Note that concanamycin A–treated cells contained large vacuolar aggregates. A = autophagosome-like vesicle; M = mitochondria VA = vacuolar aggregate; Ve = single membrane vesicle. Scale bars: (Low Mag.) = 20 µm; (High Mag.) = 1 µm.

Window stage leaves exposed to autophagy modulators were also examined using TEM (**Figure 6**). In the mock control treatment group, some autophagosome-like structures were observed in NPCD and LPCD stage cells (A, **Figure 6**), along with numerous single-membrane vesicles that varied in size and shape (Ve, **Figure 6**). Late-PCD stage cells had vacuolar aggregates (VA), whereas NPCD cells had little to no material in the vacuole (**Figure 6**). Compared to the mock control treatment group, 5 µM rapamycin-treated cells had the highest number of visible small, single-membrane vesicles (Ve) and larger vacuolar aggregates (VA, **Figure 6**). Similarly, 1 µM concanamycin had noticeably larger vacuolar aggregates compared to the mock control treatment group (VA, **Figure 6**) and had many single-membrane vesicles (Ve, **Figure 6**).

### Lace Plant Novel Cell Death Assay

**Figure 7:**
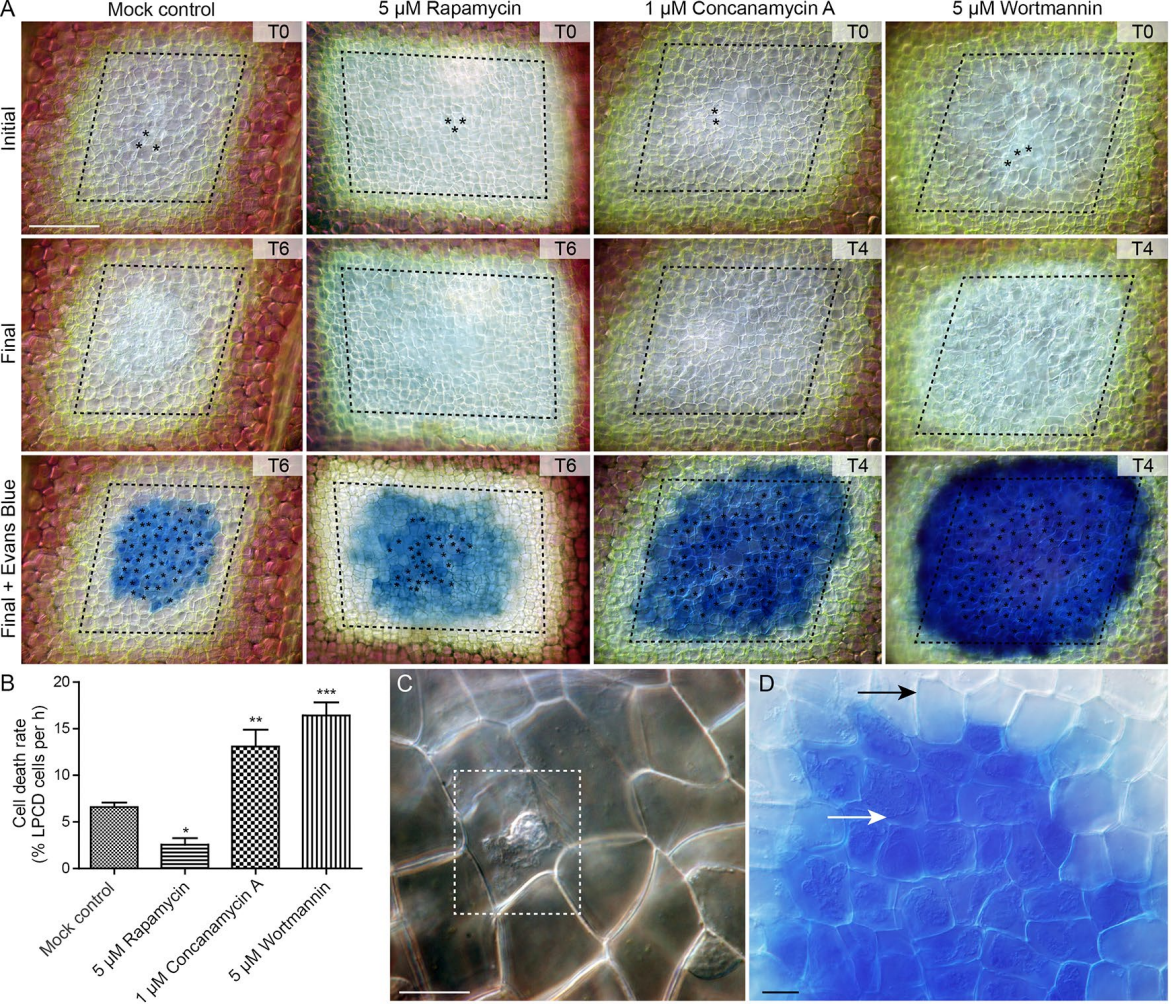
Cell death assay. **(A)** Initial and final micrographs of window stage leaves. Evans blue staining was performed at the end of the experiments to facilitate the final scoring of cell death in epidermal cells (asterisks). Treatments included 0.05% DMSO (mock control), 5 µM rapamycin, 1 µM concanamycin A, or 5 µM wortmannin. T = time, rounded to the nearest h. **(B)** The rate of cell death was calculated as the % of late programmed cell death (LPCD) cells that died per hour. **(C)** High magnification view of unstained dead epidermal cell (dashed box) in a window stage leaf. Evans blue staining **(D)** was used to facilitate quantification of dead cells in the epidermis (white arrow) at the end of experiments. Black arrow = living cell with intact plasma membrane. One-way analysis of variance, Dunnett multiple-comparisons test, n ≥ 6 leaves from individual plants; ***P < 0.001; **P < 0.01; *P < 0.05). Error bars represent standard error. Scale bars: A = 150 µm; C and D = 5 µm. (For more details, see **Supplementary File 5**.)

To assess the effect of autophagy on cell death rate during lace plant development, a novel live cell imaging assay was developed (**Figure 7**; see also **Supplementary File 5**). Window stage areoles with one to three dead cells in the epidermal layer (asterisks, **Figure 7A**, and dashed box, **Figure 7C**) were selected to ensure that PCD was synchronized in the samples. Continuous videos were captured for the control, 5 µM rapamycin, 1 µM concanamycin A, and 5 µM wortmannin treatment groups (**Figure 7A**). At the end of experiments, DIC micrographs were taken (Final, **Figure 7A**) prior to Evans blue staining (Final + Evans blue; **Figures 7A, D**), which was done to facilitate scoring of dead cells in the epidermal layer. The rate of cell death (% of LPCD cells per hour) was determined for each treatment group (**Figure 7B**). Leaves of the mock control treatment group had a mean cell death rate of 6.59 ± 0.48 (% LPCD cells per hour). The 5 µM rapamycin treatment significantly reduced the rate of cell death to 2.56 ± 0.70 (% LPCD cells per hour). Conversely, the 1 µM concanamycin A and 5 µM wortmannin treatments significantly increased the rate of cell death rates in relation to the control to 13.10 ± 1.80 and 16.42 ± 1.39 (% LPCD cells per hour), respectively.

### Autophagy Modulation and the Formation of Perforations

**Figure 8:**
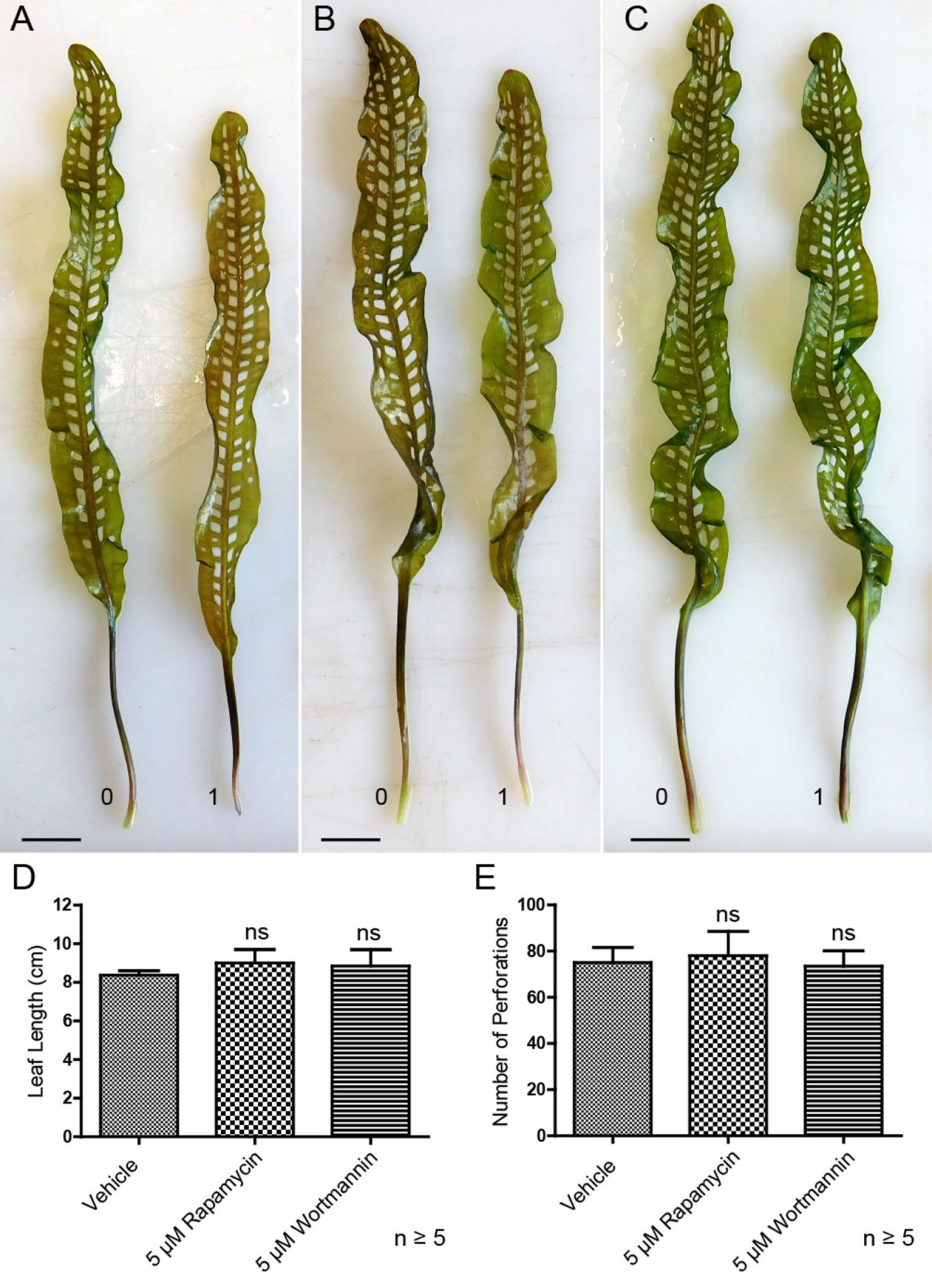
Autophagy modulation *in vivo*. Representative leaves for the DMSO control **(A)**, 5 µM rapamycin **(B)**, and 5 µM wortmannin **(C)** treatment groups. Leaf 0 represents the last to develop prior to treatment application, and leaf 1 is the first to develop afterward. **(D)** Mean leaf lengths of mature leaves posttreatment. **(E)** The number of perforations in mature leaves following treatment. One-way analysis of variance, Dunnett multiple-comparisons test, n ≥ 5 plants, ns = non-significant, *P* > 0.05). Error bars represent standard error. Scale bars: 0.5 cm.

Lace plants grown in axenic cultures were treated with autophagy modulators including rapamycin and wortmannin to determine their effects on the formation of perforations (**Figure 8**). Mock control treatment group plants (**Figure 8A**) produced leaves with an average length of 8.37 ± 0.23 cm (**Figure 8D**) and developed 75 ± 6.66 perforations (**Figure 8E**). The length of leaves of rapamycin-treated plants (**Figure 8B**) were not significantly different (9 ± 0.70 cm), as well as the number of perforations formed (78 ± 10.58). Wortmannin-treated plants did not differ from the mock control treatment group in terms of perforations (73.43 ± 6.70) per leaf and leaf length (8.85 ± 0.84 cm). *In vivo* experiments were also carried out with 1 µM AZD, but similar to autophagy enhancement with rapamycin, there was no observable response in terms of formation of perforations and leaf lengths (data not shown).

## Discussion

Autophagy is a critical life process that allows for the degradation and repurposing of cytoplasmic constituents (Feng et al., 2014). In eukaryotes, autophagy plays a central role in development and is implicated in numerous human diseases including, but not limited to, cancer, diabetes, and neurodegeneration (Tsujimoto and Shimizu, 2005; Choi et al., 2013; Jiang and Mizushima, 2014). According to Mariño et al. (2014), there is a considerable interplay between the autophagy and PCD signaling pathways, and the modulation of autophagy can have antagonistic effects depending on the experimental conditions (Minina et al., 2014). In plants, autophagy can be induced by exposure to various abiotic stresses such as starvation, exposure to saline conditions, drought, and hydrogen peroxide (Liu and Bassham, 2012). Autophagy has also been implicated in PCD following the invasion of pathogens during the hypersensitive response, as well as developmental processes ranging from embryogenesis to senescence (Liu and Bassham, 2012; Minina et al., 2013; Hofius et al., 2018). Because of the involvement of autophagy in various plant PCD systems, we investigated the extent of its involvement during lace plant leaf development.

### Autophagy and Lace Plant PCD

ATG8 and acridine orange positive puncta were observed in both healthy NPCD cells and dying cells, which indicates that autophagy occurs as part of normal homeostasis and during cellular degradation, respectively. A recent study showed that dying lace plant cells accumulate high levels of reactive oxygen species (ROS; Dauphinee et al., 2017). Autophagy is induced from various forms of stress including ROS, which may account for LPCD cells containing more ATG8-positive and acridine orange puncta compared to healthy NPCD cells. Our TEM observations of the lace plant PCD gradient confirmed the presence of double-membrane–bound autophagosome-like structures in NPCD and PCD cells (A, **Figure 6**). Additionally, numerous single-membrane vesicles (Ve, **Figure 6**) of varying shapes and sizes were found, having a similar appearance to the provacuoles formed during cellular degradation in the embryos of Norway spruce (*Picea abies*; Filonova et al., 2000; Minina et al., 2013) or autolysosomes in BY-2 cells (Takatsuka et al., 2017). The red fluorescence in LPCD cells stained with acridine orange suggests that the puncta are acidic vesicles and may serve a similar function to autolysosomes. Like acridine orange, MDC accumulates in acidic compartments (Klionsky et al., 2016), and previous work in the lace plant showed that NPCD cells contain ATG8 and MDC-positive puncta (Mishra et al., 2017). A general increase in the size of the vacuoles was observed as degradation of the cytoplasm advanced throughout PCD (**Figures 2** and **6**), which is commonly observed during developmental PCD in plants (Liu and Bassham, 2012). Vacuolar aggregates in lace plant cells that comprised electron-dense, degraded organelle material were also seen to increase in size as PCD progressed; this was consistent with preliminary lace plant TEM observations (Wertman et al., 2012).

### Modulation of Autophagy in Lace Plants

Live cell and TEM observations showed that commercially available autophagy modulators are effective in lace plant cells. The effects of autophagy modulation were most pronounced in NPCD cells, where autophagosome-like vesicles were moving quickly and clearly visible using time-lapse live cell imaging (**Figure 5**; **Supplementary File 3**). Interestingly, wortmannin-treated window stage NPCD cells contained larger, slow-moving vesicles that appeared to have organelles within them. Similar vesicles were observed following cell death induction from high pH conditions (Dauphinee et al., 2014), suggesting these organelle-containing vesicles may form under stressful conditions in lace plant cells. The novel cell death assay presented here also highlights the advantages of using the lace plant model to study autophagy (**Figure 7**; **Supplementary File 5**). Our cell death assay results indicate that enhancement of autophagy led to prolonged lifespan in LPCD cells, and conversely, the inhibition of autophagy led to a greater rate of cell death. Although the autophagy-modulating compounds had effects at the cellular level, there was no observed effect *in vivo* on lace plant leaf development even at higher concentrations (data not shown). Therefore, autophagy modulation itself is not enough to significantly influence the formation of perforations or lace plant leaf development under optimal growth conditions. However, future experiments should be done to determine how modulation may affect lace plant development under stressful conditions. In terms of ultrastructural observations following autophagy modulation, the 5 µM rapamycin treatment generated a visible increase in vesicles that appeared to contain more electron-dense material compared to the control. Concanamycin A–treated specimens also had an abundance of vesicles and had the largest vacuolar aggregates, which was evident *via* TEM. Autophagic bodies found within the vacuoles of *Arabidopsis* roots (Merkulova et al., 2014) have a similar appearance at the light microscopy level to the vacuolar aggregates detailed here in the lace plant.

### Conclusions and Future Work

Lace plant leaves provide an excellent system to study the role of autophagy on cell death or survival since both types of cells (NPCD and PCD) are simultaneously present within an areole of a window stage leaf. Although autophagy modulation led to delayed or enhanced cell death rates toward the later stages of PCD, our results indicate that autophagy is predominantly a survival mechanism in the lace plant, and we did not observe clear evidence for its direct involvement in the induction of developmental PCD under normal circumstances. The lace plant presents a tractable model for studying the core autophagy machinery *in planta*; however, more advanced tools are necessary to better understand this biochemical pathway. Future aims include genetic modification to create GFP–ATG8 lines and the establishment of autophagy-deficient mutants, which would be invaluable tools to understand autophagy in the emerging lace plant model system.

## Data Availability Statement

The raw data supporting the conclusions of this manuscript will be made available by the authors, without undue reservation, to any qualified researcher.

## Author Contributions

AD carried out experiments and wrote the manuscript. GD and AR contributed to whole plant and live cell imaging experiments. MF contributed to cell death assay experiments. CL participated in manuscript revisions. AG secured funding for the study, designed the experiments, contributed to manuscript revisions, and supervised all experimental work. All authors read and approved the final version.

## Funding

This work was supported by AG’s NSERC Discovery Grant (# 2017-04299) and Accelerator Supplements (# 2017-507825).

## Conflict of Interest

The authors declare that the research was conducted in the absence of any commercial or financial relationships that could be construed as a potential conflict of interest.

## Supplementary Material

Supplemental File 1 Acridine orange staining in window stage leaves. Non-programmed cell death (NPCD) and late-programmed cell death (LPCD) stage cells were stained with acridine orange and observed using confocal microscopy. Acridine orange was excited at 488 nm and detected at 525/25 nm (green) and 595/50 nm (magenta). Actual acquisition time: 5 min. Scale bar: 15 µm. Video 1.MP4.

Supplemental File 2 Acridine orange and monodansylcadaverine dual staining in window stage leaves. Non-programmed cell death (NPCD) were stained and observed using confocal microscopy. Fluorescent laser scanning confocal micrographs represent maximum intensity projections of z-stack acquisitions. Corresponding differential interference contrast (DIC) is taken from a single representative focal plane within the z-stack. Excitation with 405 and 488 nm light was used for MDC and acridine orange stains, respectively. Fluorescence emission was detected at 450/35 nm (cyan), 525/25 nm (green), and 595/50 nm (magenta). Scale bar: 20 µm. Image 1. TIF.

Supplemental File 3 Live cell imaging time-lapse videos of non-programmed cell death (NPCD) window stage cells. Treatments include a negative control, mock control treatment group (DMSO), 1 µM AZD 8055, 5 µM rapamycin, 1 µM concanamycin A or 5 µM wortmannin. Negative control leaves were scanned immediately after removed from culture and all other groups had a 16-h starvation period in distilled water prior to treatment application. Click on individual videos to play/pause. Actual acquisition time: 5 min. Scale bar: 20 µm. For additional information see **Figure 4**. Presentation 1.PPTX (video files).

Supplemental File 4 Live cell imaging time-lapse videos of late-programmed cell death (LPCD) window stage cells. Treatments include a negative control, mock control treatment group (DMSO), 1 µM AZD 8055, 5 µM rapamycin, 1 µM concanamycin A or 5 µM wortmannin. Negative control leaves were scanned immediately after removed from culture and all other groups had a 16-h starvation period in distilled water prior to treatment application. Click on individual videos to play/pause. Actual acquisition time: 5 min. Scale bar: 20 µm. For additional information see **Figure 4**. Presentation 2.PPTX (video files).

Supplemental File 5 Cell death assay. Mock control treatment group (DMSO), 5 µM rapamycin, 1 µM concanamycin and 5 µM wortmannin-treated window stage leaves. Actual acquisition time: 4h (Concanamycin A, Wortmannin) – 6h (Control, Rapamycin). Scale bar:100 µm. Video 2.MP4.
