# Supplementary figures and images for: The Function of Autophagy in Lace Plant Programmed Cell Death
Source: Front Plant Sci. 2019 Oct 22;10:1198. doi: 10.3389/fpls.2019.01198 (PMC6817616; doi:10.3389/fpls.2019.01198)

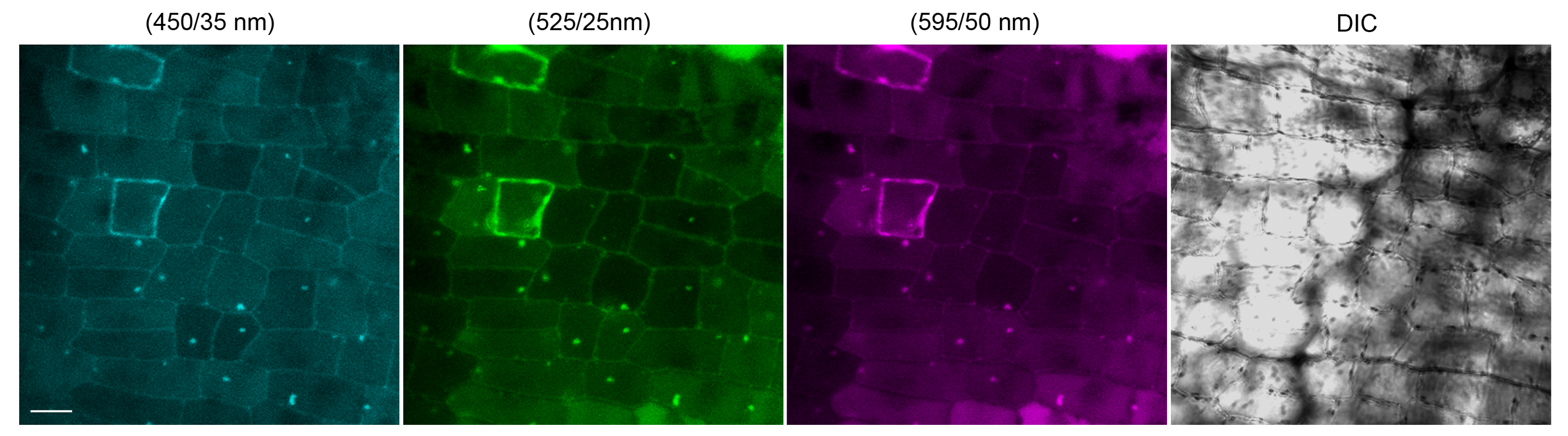

Supplement: Supplemental File 2 — Acridine orange and monodansylcadaverine dual staining in window stage leaves. Non-programmed cell death (NPCD) were stained and observed using confocal microscopy. Fluorescent laser scanning confocal micrographs represent maximum intensity projections of z-stack acquisitions. Corresponding differential interference contrast (DIC) is taken from a single representative focal plane within the z-stack. Excitation with 405 and 488 nm light was used for MDC and acridine orange stains, respectively. Fluorescence emission was detected at 450/35 nm (cyan), 525/25 nm (green), and 595/50 nm (magenta). Scale bar: 20 µm. Image 1. TIF. [file Image_1.tif]

## Slide 1
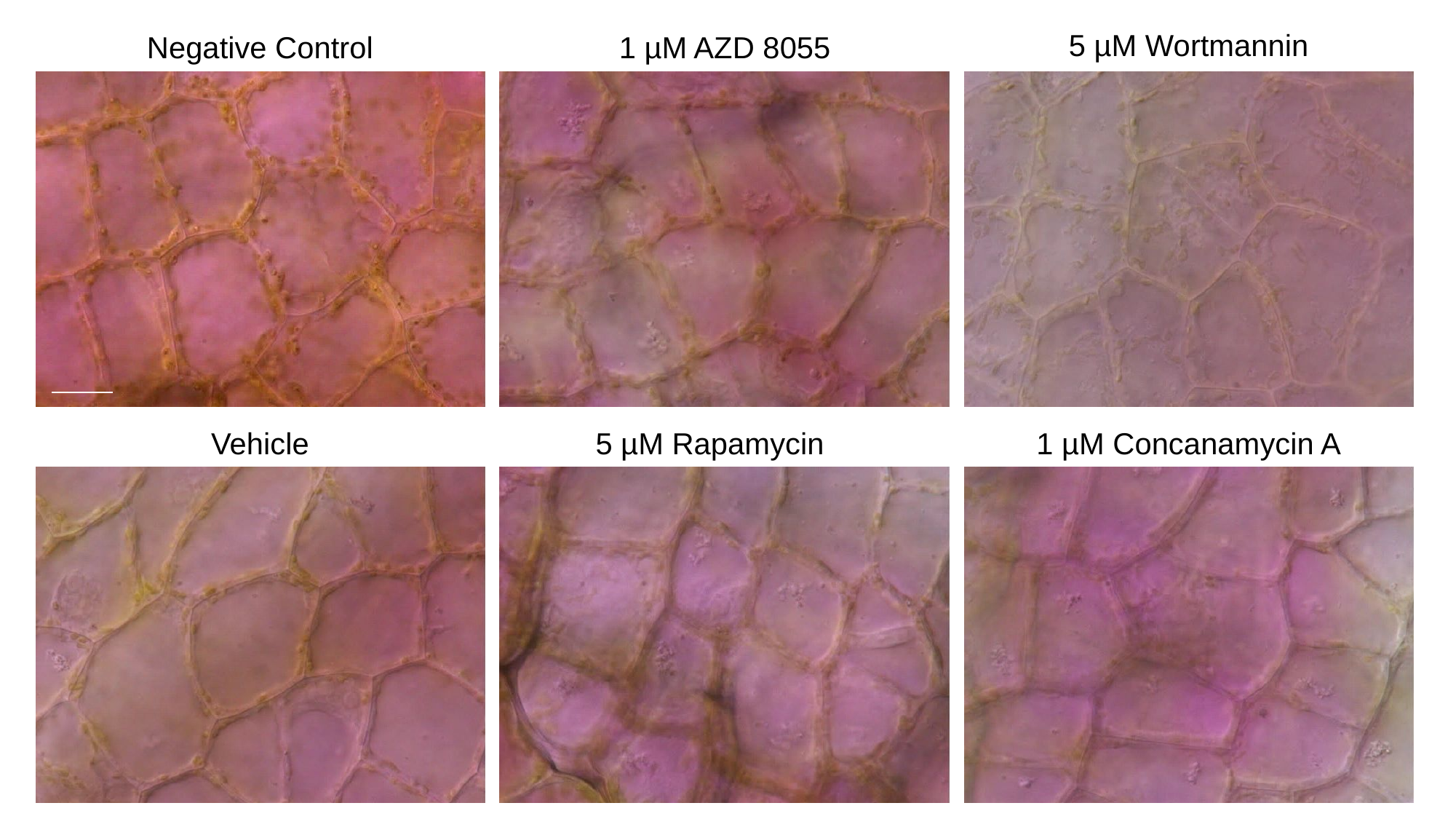

5 µM Wortmannin
Negative Control
1 µM AZD 8055
Vehicle
5 µM Rapamycin
1 µM Concanamycin A

Supplement: Supplemental File 3 — Live cell imaging time-lapse videos of non-programmed cell death (NPCD) window stage cells. Treatments include a negative control, mock control treatment group (DMSO), 1 µM AZD 8055, 5 µM rapamycin, 1 µM concanamycin A or 5 µM wortmannin. Negative control leaves were scanned immediately after removed from culture and all other groups had a 16-h starvation period in distilled water prior to treatment application. Click on individual videos to play/pause. Actual acquisition time: 5 min. Scale bar: 20 µm. For additional information see Figure 4 . Presentation 1.PPTX (video files). [file Presentation_1.pptx]

## Slide 1
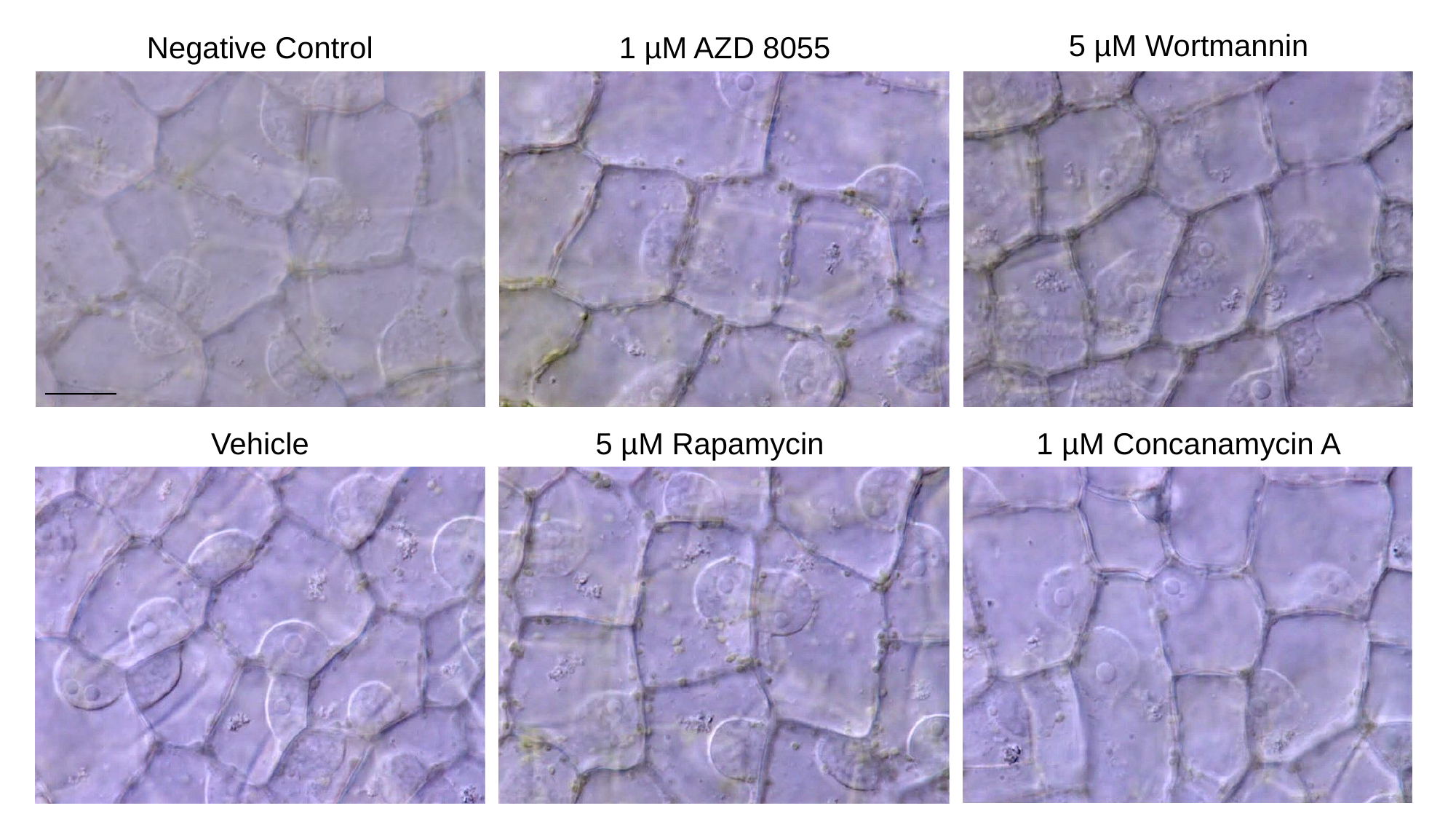

5 µM Wortmannin
Negative Control
1 µM AZD 8055
Vehicle
5 µM Rapamycin
1 µM Concanamycin A

Supplement: Supplemental File 4 — Live cell imaging time-lapse videos of late-programmed cell death (LPCD) window stage cells. Treatments include a negative control, mock control treatment group (DMSO), 1 µM AZD 8055, 5 µM rapamycin, 1 µM concanamycin A or 5 µM wortmannin. Negative control leaves were scanned immediately after removed from culture and all other groups had a 16-h starvation period in distilled water prior to treatment application. Click on individual videos to play/pause. Actual acquisition time: 5 min. Scale bar: 20 µm. For additional information see Figure 4 . Presentation 2.PPTX (video files). [file Presentation_2.pptx]
